# Diagnosis and Management of Vitamin D Dependent Rickets

**DOI:** 10.3389/fped.2020.00315

**Published:** 2020-06-12

**Authors:** Michael A. Levine

**Affiliations:** Center for Bone Health and Division of Endocrinology and Diabetes, The Children's Hospital of Philadelphia and University of Pennsylvania Perelman School of Medicine, Philadelphia, PA, United States

**Keywords:** rickets, genetics, Vitamin D, treatment, hypocalcemia

## Abstract

The term “vitamin D dependent rickets” describes a group of genetic disorders that are characterized by early-onset rickets due to the inability to maintain adequate concentrations of active forms of vitamin D or a failure to respond fully to activated vitamin D. Although the term is now admittedly a pathophysiological misnomer, there remains clinical relevance for its continued use, as patients have a lifelong “dependency” on administration of specialized regimens of vitamin D replacement. This review provides an update on the molecular bases for the three forms of vitamin D dependent rickets, and summarizes current protocols for management of affected subjects.

## Introduction

The recognition that genetic defects in vitamin D activation or responsiveness could cause rickets evolved from detailed clinical and biochemical studies that identified the critical role that vitamin D played in ensuring normal bone and mineral metabolism. Historically, rickets has been considered a manifestation of poor socioeconomic status and a diet that failed to provide adequate amounts of vitamin D and/or calcium (1, 2). Early studies revealed that rickets was caused by combined nutritional deficiency and a sunless environment, and could be successfully treated with cod liver oil (3). A chemical basis for the therapeutic effectiveness of cod liver oil was first proposed in 1919 by Edward Mellanby (4), and subsequently biochemist Elmer McCollum, working with Johns Hopkins pediatrician John Howland, and later with doctors Edwards A. Park and Paul G. Shipley, showed the anti-rachitic substance to be a novel vitamin, which was then named vitamin “D” using the next free letter of the alphabet (5). Parallel work during this time by many investigators (6, 7) led ultimately to the observation that ultraviolet light exerted a systemic effect to cure rickets (7). Alfred Hess and Lester Unger later showed that sunlight was anti-rachatic, an observation that led Harriette Chick to pursue the controlled studies of sunlight and cod liver oil that confirmed the effectiveness of each to prevent rickets. These studies led ultimately to commerical development of vitamin D, providing abundant, inexpensive supplies of vitamin D that enabled widespread prevention and treatment of rickets.

The subsequent use of vitamin D (calciferol) for prevention and treatment of rickets and osteomalacia led to marked reductions in their prevalence and clinical burden. Nevertheless, some patients did not respond to the usual doses of calciferols, which led to the recognition of genetic and clinical disorders that impaired activation and/or responsiveness of target tissues to active metabolites of vitamin D. In 1937, Albright reported studies of a child with rickets who failed to respond to the usual doses of vitamin D, and suggested that the basis for the rickets was a hereditary resistance to the actions of calciferols (8). Hereditary cases of rickets that were resistant to calciferols were subsequently recognized, but most patients had biochemical findings that differed from those of nutritional deficiency of calciferol, with normal serum calcium levels but reduced serum levels of phosphorus and impaired net renal tubular reabsorption of phosphate anion. This condition, originally termed familial vitamin D-resistant rickets, was subsequent named X-linked hypophosphatemia (XLH) to acknowledge its X-linked dominant inheritance and the role of primary hypophosphatemia in the development of rickets and osteomalacia. Today we recognize that there are multiple forms of inherited hypophosphatemic rickets in addition to XLH. Many of these additional forms share in common with XLH the presence of excess circulating levels of FGF23, a phosphotonin that accounts for decreased renal tubular reabsorption of phosphate and hypophosphatemia (9).

True defects in vitamin D responsiveness were first identified as a cause of rickets in 1961, when Prader and colleagues (10) described a distinctive form of hereditary rickets characterized by hypocalcemia and defective bone mineralization, and which did not respond to conventional vitamin D therapy. This disorder was termed pseudodeficiency rickets. This review provides an overview of the growing number of gene defects that interfere with vitamin D action and cause various forms of vitamin D-dependent rickets (VDDR).

## Vitamin D Homeostasis and Rickets

**Figure 1:**
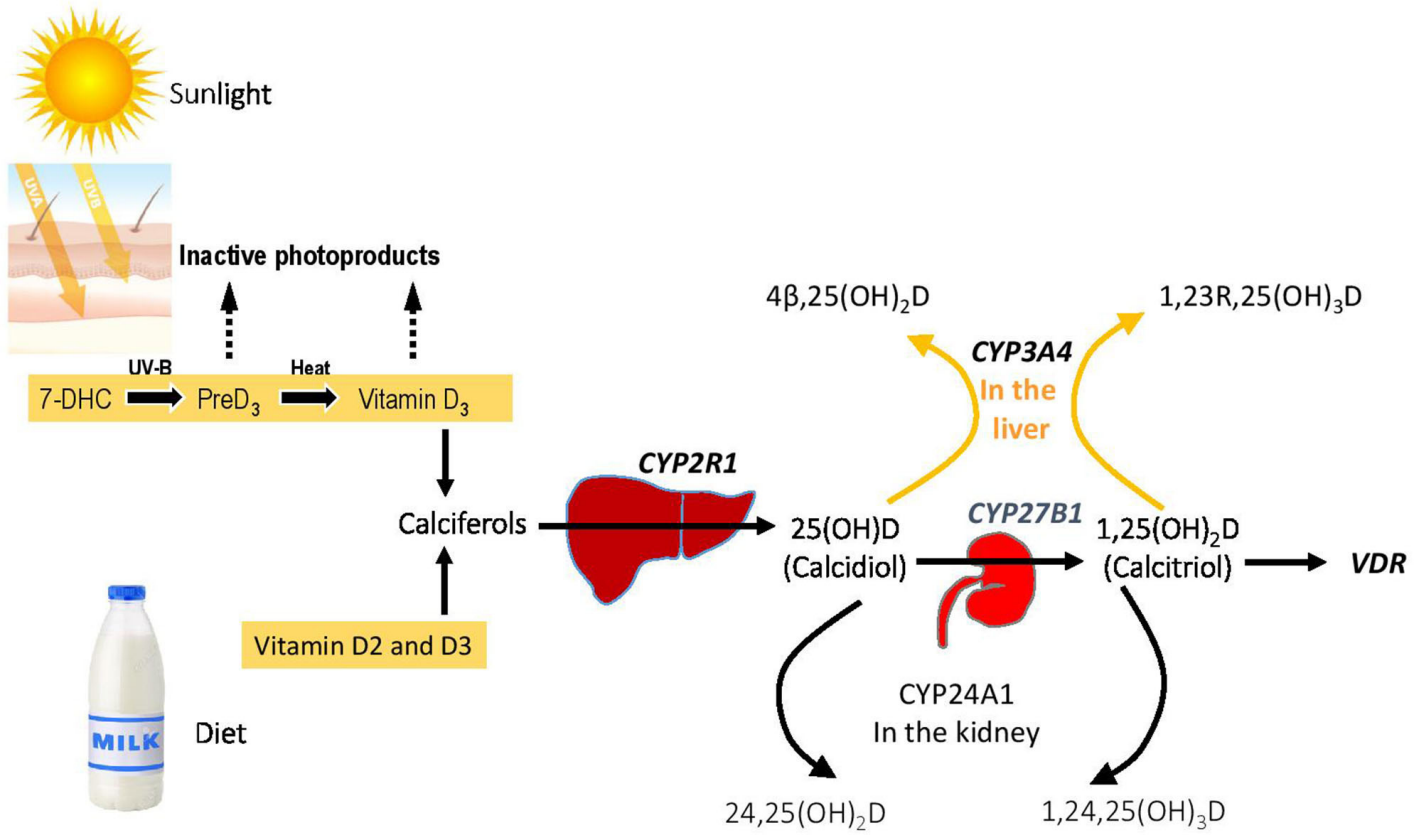
Vitamin D homeostasis and genetic blocks in VDDR. The figure shows the overall metabolic control of vitamin D homeostasis, with the genes involved in various forms of VDDR shown in bold.

Cholecalciferol (vitamin D_3_) is synthesized in a reaction that begins with the opening of the B-ring of 7-dehydrocholesterol to form previtamin D3, which (Figure 1). This reaction is driven by ultraviolet radiation (UVB), typically from sunlight, that reaches the basal layers of the epidermis (11). Previtamin D3 is thermally labile, and it undergoes a temperature-dependent isomerization to vitamin D3 over a period of 3 days. Melanin content of skin, aging, and physical barriers such as clothing or UV sunblock can reduce the amount of cholecalciferol produced in response to UV irradiation (12, 13). Ergocalciferol (termed vitamin D_2_) is synthesized by opening the B-ring of ergosterol, a sterol present in yeast, fungi, and protozoa. Activation of the parent calciferols cholecalciferol and ergocalciferol requires two hydroxylations. The first modification is 25-hydroxylation, which occurs principally in liver microsomes by the P450 enzyme CYP2R1 (14). Plasma 25-hydroxyvitamin D [25(OH)D] is the most abundant circulating vitamin D metabolite and provides the best index of vitamin D status. To gain full potency, 25 (OH)D undergoes 1α-hydroxylation to 1,25 (OH)_2_D by the mitochondrial cytochrome P450 by CYP27B1 in kidney proximal tubule cells. Renal CYP27B1 is tightly regulated, and expression and activity of the enzyme are induced by PTH and suppressed by FGF23. Vitamin D metabolites 25(OH)D and 1, 25(OH)2D may also be modified by the renal cytochrome P450 CYP24A1, which converts these molecules into inactive 24-hydroxylated products. A secondary pathway for inactivation of vitamin D metabolites is provided by CYP3A4, which is highly expressed in liver and small intestine and which can biotransform a variety of compounds that have clinical importance, including many drugs, steroids, xenobiotics, and carcinogens.

Finally, biological activity of 1,25 (OH)_2_D involves binding of the hormone to two different cellular receptors. Genomic actions are mediated by interaction with a high-affinity intracellular nuclear receptor termed the VDR that is analogous to the receptors for other steroid hormones (15), whereas non-genomic actions are mediated by binding of 1,25(OH)_2_D to a presumptive plasma membrane receptor that appears to be identical to classical intranuclear steroid hormone receptors (16). Ligand-activation of this signaling pathway results in stimulation of MAP-kinase and involves crosstalk with the nuclear VDR [see (17) for review] as well as interaction of membrane receptors with other proteins (e.g., ion channels) or signal transduction pathways (e.g., protein kinase C, cAMP, Ca^++^, etc.). Some reports suggest that the membrane-bound vitamin D receptor is important for both fracture healing and chondrocyte maturation (18, 19).

Nearly all features of vitamin D deficiency represent a direct or indirect consequence of the loss of vitamin D action on duodenal transport of calcium and to a lesser degree on impaired mobilization of calcium from skeletal stores. Hypocalcemia, and to some degree calciferol deficiency, results in increased section of PTH from the parathyroid glands (i.e., secondary hyperparathyroidism), which leads to decreased reabsorption of phosphate and bicarbonate in the proximal renal tubule, resulting in hypophosphatemia and hyperchloremic acidosis, respectively. Hypocalcemia and hypophosphatemia collectively reduce the rate of mineralization of bone matrix. Reduced serum levels of calcium and/or phosphorus can produce osteomalacia, as the low concentrations of these minerals are insufficient to induce or propagate hydroxyapatite cystral deposition in the skeleton. By contrast, rickets arises as a result of a growth plate defect that in which hypertrophic chondrocytes fail to undergo caspase 9-mediated apoptosis due to reduced concentrations of extracellular phosphate (20). This effect of hypophosphatemia provides a final common pathway that unifies the growth plate defect of calciopenic forms of rickets (i.e., rickets due to deficiency of vitamin D and/or calcium) with that of hypophosphatemic forms of rickets. The enlarged and disorganized chondrocytes weaken the growth plate and fail to secrete signals that are required for normal replacement of the cartilege template by endochondral bone. PTH can also act directly on bone to increase osteoclastic reabsorption, but in the absence of adequate 1,2(OH)2D action the release of calcium and phosphorus from bone is impaired and cannot fully reverse hypocalcemia or hypophosphatemia.

**Figure 2:**
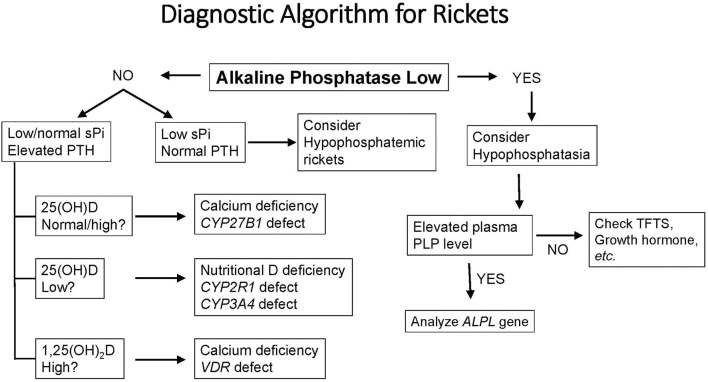
A diagnostic algorithm for evaluation of a child with radiological or clinical features of rickets. See text for complete description of biochemical and clinical features of each form of rickets. PLP, Pyridoxal 5′-phosphate, the metabolically active form of vitamin B6; sPi, serum phosphorus; TFTs, thyroid function tests; PLP, pyridoxal 5′ phosphate (vitamin B6); ALPL, the gene for tissue non-specific alkaline phosphatase.

Because the appearance of the growth plate in children with rickets can resemble that of subjects with hypophosphatasia or metaphyseal dysplasias, a reasonable first step in diagnosis is to determine the serum level of alkaline phosphatase (Figure 2). Using age- and sex-related references ranges, alkaline phosphatase activity will usually be elevated in rickets, normal in metaphyseal dysplasia, and low in hypophosphatasia. Subjects who have hypophosphatemia and normal serum levels of PTH, calcium, and 25(OH)D are likely to have phosphopenic rickets; a normal or elevated tubular reabsorption of phosphorus (TRP) will be found in subjects with inadequate intake or absorption of phosphorus. By contrast, patients with XLH and other genetic or acquired causes of renal phosphate wasting will have reduced TRP values. Subjects with calciopenic rickets will have elevated serum levels of PTH, which depress the TRP and cause hypophosphatemia. These subjects have either inadequate intake of calcium and/or vitamin D or genetic defects that impair vitamin D activation or responses (i.e., VDDR). Serum levels of calcium will be normal or decreased based the on the severity of the underlying condition.

## Vitamin D Dependent Rickets: General Principles

There are three broad categories of VDDR (Figure 1, Table 1). The first is classified as VDDR type 1 (VDDR1), and represents a failure to fully activate calciferols due to the inability to generate either 25(OH)D (VDDR1b) or 1,25(OH)_2_D (VDDR1a). The second category is VDDR2, and is characterized by resistance to 1,25(OH)_2_D owing to mutations in the VDR (VDDR2a) or the presence of a nuclear ribonucleoprotein that interferes with the vitamin D receptor-DNA interaction (VDDR2b). The third category is VDDR3, which results from excessive inactivation of vitamin D metabolites. VDDR shares many clinical and biochemical characteristics with vitamin D deficiency rickets, and Figure 2 provides a suggested diagnostic algorithm. Table 1 outlines the biochemical features of the various forms of VDDR.

**Table 1:** Vitamin D-dependent rickets.

**Type**	**25(OH)D**	**1,25(OH)_**2**_D**	**PTH**	**Inheritance**	**Gene defect (OMIM)**
VDDR1A	N/I	D	I	A.R.	CYP27B1 (264700)
VDDR1B	D	D	I	A.R.	CYP2R1 (600081)
VDDR2A	N/I	N/I	I	A.R.	VDR (277440)
VDDR2B	N/I	N/I	I	A.R.	Unknown (600785)
VDDR3	D	D	I	A.D.	CYP3A4 (124010)

*VDDR, vitamin D-dependent rickets, N, normal; I, increased, D, decreased; PTH, parathyroid hormone*.

Because hypophosphatemia is the unifying basis for all forms of rickets (20), the general principle of VDDR management is to maintain the serum calcium level in the midnormal range to achieve a normal serum level of PTH. Normalization of the serum PTH concentration will restore normal renal TRP, with consequent correction of hypophosphatemia. Persistently elevated levels of PTH may lead to hyperparathyroid bone disease, while suppressed levels of PTH are usually associated with hypercalciuria. Despite this common pathophysiology, the three forms of VDDR show important differences in the biochemical profile of circulating vitamin D metabolites, the therapeutic approach, and in the genetic defect in vitamin D metabolism or action (Figure 2, Table 1).

Patients should be monitored regularly to detect potential asymptomatic hypo- or hypercalcemia, to ensure that growth is normal, to observe changes in the shape or structure of the bones, and to determine whether rickets is active. Serum (calcium, phosphorus, PTH, alkaline phosphatase, creatinine, and appropriate vitamin D metabolites) and urinary (24-h urine calcium or random urine calcium/creatinine ratio) biochemistries should be measured every 3–6 months. In order to monitor for renal calcification, a renal ultrasound examination should be performed every 1–2 years, and more frequently if there is evidence of hypercalciuria. Rachitic activity can be assessed by annual radiographs of a wrist and knee.

## Vitamin D-Dependent Rickets Type 1a (VDDR1a)

Patients with VDDR1a (OMIM 264700) are unable to convert 25(OH)D to 1,25(OH)_2_D owing to biallelic mutations in the *CYP27B1* gene on chromosome 12q13.3 that encodes 25-hydroxyvitamin D_3_-1α-hydroxylase. Therefore, VDDR1a represents a selective and simple deficiency of 1,25(OH)_2_D. The complete response of the original index cases to very high doses of vitamin D suggested vitamin D deficiency, but the persistent need for unusually high doses of vitamin D to maintain a remission signified a vitamin D dependency. VDDR1a occurs with greatest frequency in the French-Canadian population (21).

Affected subjects appear normal at birth but problems related to impaired vitamin D activity become obvious at 2–24 months. Hypotonia, irritability, tetany or seizures, and failure to thrive, are typical in the first few months of life. If diagnosed later, fractures, and typical skeletal features of rickets (e.g., frontal bossing, long-bone deformities, and rib cage abnormalities) as well as impaired growth are important clinical features. Biochemical studies show hypocalcemia, hypophosphatemia, and elevated serum levels of alkaline phosphatase and parathyroid hormone. Plasma concentrations of 1,25(OH)_2_D are low or even undetectable. By contrast, plasma concentrations of 25 (OH)D are normal or increased, likely reflecting the effects of both vitamin D supplementation and decreased clearance of 25 (OH)D due to lack of induction of CYP24A1 by the absence of 1,25(OH)2D. If untreated, there is progressive skeletal deformity and short stature. With adequate treatment there is healing of rickets and normal growth.

All available calciferol analogs have been successfully used to treat patients with VDDR1a (22–25), but activated forms of vitamin D, such as calcitriol, offer significant advantages: (1) 1,25 (OH)_2_D_3_ is the deficient hormone and therefore, physiologic doses of calcitriol are effective; (2) Due to the relatively short half-life of calcitriol, there is a rapid onset of action and unintentional hypercalcemia resolves within days of discontinuation of the drug; and (3) calcitriol is available for oral administration as either a small capsule or a suspension and can also be given intravenously. Calcitriol is usually administered twice per day owing to its short half-life. Treatment with 1α-cholecalciferol is similarly effective as this metabolite also overcomes the enzymatic block, and due to its longer half-life than calcitriol, it can be administered once daily. Because high concentrations of 25(OH)D can bind and activate the VDR, it is also possible to use parent vitamin D (ergocalciferol or cholecalciferol) or 25(OH)-vitamin D (calcifediol), but these analogs must be administered at pharmacologic doses, which carries a greater risk of inducing hypercalcemia. During successful treatment with vitamin D or calcifediol, serum levels of 25(OH)D levels will be very high (e.g., 150–250 ng/mL), but the plasma concentration of 1,25 (OH)_2_D can remain low or even undetectable (26, 27). Because of the long half-life of 25(OH)D, these drugs afford once-daily dosing. During the first 3–6 months of treatment, patients should be given calciferols in doses 2–5 times those expected for long-term maintenance (Table 2), as the undermineralized skeleton requires unusually large amounts of calcium. Calcium supplements (50 mg/kg of elemental calcium per day for children) should also be given during initial treatment to prevent worsening of hypocalcemia due to the “hungry bones” phenomenon that occurs with remineralization of the skeleton.

**Table 2:** Suggested calciferol doses for maintenance treatment of patients with VDDR.

	**VDDR1A**	**VDDR1B**	**VDDR2**	**VDDR3**
	**(μg per day)**	**(μg per day)**	**(μg per day)**	**(μg per day)**
Vitamin D3 or D2	NI	100–200	125–1,000?*	**1,000 to?**
Calcifediol	NI	**20–50**	20–200*	50 to?
Calcitriol	**0.3–2**	0.3–2	**5–60†**	1 to?
1α (OH)D	**0.5–3**	0.5–3	**5–60†**	2 to?

*Dose requirements are uncorrected for body weight and are similar in children and adults. In all cases, supplemental calcium is recommended as described in text. The preferred form of calciferol is noted in bold for each disorder. NI, not indicated*.

* *Patients with milder grades of resistance to 1,25(OH)2D (usually with normal hair) often can respond to analogs requiring 1-hydroxylation. Maximal useful doses have not been defined. Serum 1,25(OH)2D must be maintained in the range of 200–1,000 pg/mL*.

† *Maximal does are limited only by cost and patient acceptance; some patients have shown no response to maximal doses tested*.

With successful therapy, the fractional absorption of calcium in the intestine remains constant and levels of calcium in the serum (and urine) can fluctuate greatly with variations in the dietary intake of calcium. It therefore makes sense to recommend calcium supplements to ensure a consistent intake of calcium. Treatment must be continued indefinitely.

## Vitamin D-Dependent Rickets Type 1b (VDDR1b)

VDDR1b (OMIM #600081) is due to mutations in *CYP2R1* that decrease expression or function of the encoded CYP2R1 enzyme, the principal 25-hydroxylase. VDDR1b is associated with hypocalcemia, secondary hyperparathyroidism and low plasma concentrations of 25(OH)D, with reduced clinical and biochemical responsiveness to conventional doses of vitamin D (28–33).

Comprehensive studies in individuals carrying the common p.L99P mutation have provided greater clinical and biochemical insights (30, 32). Subjects who were homozygous for the p.L99P mutation showed the most severe phenotype, with clinical rickets and very low serum concentrations of 25(OH)D despite extensive sun exposure. These individuals had undetectable baseline concentrations of 25(OH)D that showed negligible responses to administration of 50,000 IU of cholecalciferol or ergocalciferol (32). By contrast to other forms of VDDR, which follow a typical autosomal recessive pattern of transmission, relatives who were heterozygous for the p.L99P mutation also showed some evidence of CYP2R1 deficiency, but with less severe childhood bone disease and only modest reductions in circulating concentrations 25(OH)D concentrations. Remarkably, these abnormalities seemed to improve as the heterozygous subjects matured. Heterozygous patients also show a blunted increase of serum 25(OH)D to administration of 50,000 IU of parent vitamin D (32). These studies suggest that there is a gene dose effect on the phenotype.

The most common *CYP2R1* mutation is p.L99P. In addition, a second missense mutation, p.K242N, has also been described in a patient from Nigeria (32). Other patients from the Middle East have been reported with compound heterozygous mutations (31). In one case, there is an insertion and frameshift mutant [c.768_769insT (p.L257SfsX6)], while the other mutation is a single base change in the splice-donor site at the end of exon two (c.367 + 1, G->A). Both mutations are predicted to result in non-functional proteins, but neither molecular nor functional studies have been presented to confirm these predictions. VDDR1B has also been described in members of two unrelated families from France (33). Several affected individuals from one consanguineous family were homozygous for the p.L99P mutation. The second family contained an affected individual who was homozygous for a novel *CYP2R1* mutation, p.G42_L46delinsR.

The clinical phenotype of VDDR1B closely resembles that of VDDR1a, but with a few notable and unique features. First, the magnitude of vitamin D deficiency and the clinical severity in subjects with *CYP2R1* mutations exhibits a gene dosage effect such that the phenotype is milder in patients who carry only one defective allele. Second, the phenotype appears to improve with age, which may indicate acquisition of a vitamin D-independent mechanism(s) for intestinal absorption of calcium due to development of post-pubertal levels of sex hormones (34). Alternatively, other CYP enzymes that possess 25-hydroxylase activity may assume greater importance with maturation.

Several options for management of VDDR1B are possible. A common approach is to administer pharmacologic doses of ergocalciferol or cholecalciferol or physiological doses of calcitriol, plus supplemental calcium. The availability of calcifediol now permits a superior pharmacological approach that bypasses the defect in 25-hydroxylation. Using calcifediol to normalize plasma concentrations of 25(OH)D has the advantage of restoring physiological control of mineral metabolism, as conversion of 25(OH)D to 1,25(OH)2D will occur in response to secretion of PTH. Remarkably, Molin et al. showed successful management of one of their patients using alfacalcidol [1α(OH)D] (33). The success of this method may depend upon the specific mutation in their patient, who was homozygous for p.G42_L46delinsR. An alternative explanation is that 1α(OH)D is a better substrate than ergocalciferol or cholecalciferol for an alternate 25-hydroxylase; CYP3A4, for instance, has greater 25-hydroxylase activity for 1α(OH)D than parent calciferols (35). As for VDDR1a, serum levels of calcium and PTH should be maintained in the mid-normal range, and treatment will be required for life. Patients will require supplemental calcium.

## Vitamin D-Dependent Rickets Type 2a (VDDR2a)

VDDR2a (OMIM 277440) is due to biallelic loss-of-function mutations in the gene encoding the vitamin D receptor (VDR; 601769) on chromosome 12q13.11, and therefore represents a *bona fide* form of tissue resistance to vitamin D. As many patients with this disorder are unable to respond to any form of vitamin D, some have suggested that VDDR2 may be more appropriately described by the terms hereditary 1,25 (OH)_2_D-resistant rickets (HVDRR), hereditary resistance to 1,25 (OH)_2_D, or even pseudovitamin D-deficiency, type iia (PDDR IIA). Despite the pathophysiological relevance of these alternative names, here we shall maintain the historically conventional term, VDDR2a.

The clinical features of VDDR2a are very similar to those of patients with other forms of VDDR, but about fifty percent of patients with VDDR2a have alopecia. Patients with VDDR2a appear normal at birth and later develop features of vitamin D deficiency over the first 2–8 months of life. In addition, affected children are born with normal distribution of hair, and alopecia develops later due to failure of normal hair follicle cycling, which is dependent upon unliganded actions of the VDR. Recent studies have shown that *Vdr*-null mice develop their initial coat of hair normally, but after the first hair cycle there is impaired reinitiation of anagen (36). Lack of the VDR leads to increased expression of the hairless (*Hr*) protein, a transcription factor that regulates hair follicle cycling via interaction with unliganded *Vdr* (37). Alopecia is associated with *VDR* mutations that impair DNA binding, RXR heterodimerization, or production of the VDR, while mutations that alter VDR affinity for 1,25(OH)_2_D or disrupt coactivator interactions do not cause alopecia. Alopecia may be partial or complete, and sometimes there is sparing of the eyelashes. Alopecia appears to be associated with the most severe forms of VDDR2a, based on the very early onset of hypocalcemia and poorest response to therapy.

Biochemical studies show low serum levels of calcium and phosphorus and elevated serum concentrations of 1,25(OH)_2_D, in the range of 50–1,000 pg/mL (normal in children is 30–100 pg/mL); serum concentrations of 25(OH)D may be normal or elevated due to lack of induction of CYP24A1, which requires VDR action. Management during the first few months of life may require only administration of high doses of oral calcium (5–6 g/m^2^ body surface of elemental calcium). If oral therapy is able to restore normocalcemia and reverse secondary hyperparathyroidism it may not be necessary to initiate intravenous infusions of calcium. In some cases it may be necessary to initiate treatment with intravenous calcium to raise the serum calcium and replenish calcium deficits, after which it is reasonable to attempt a transition to oral calcium. Nevertheless, successful transition to oral calcium will require substantial doses of calcium salts.

As affected infants grow the requirement for calcium increases and vitamin D-independent intestinal absorption of calcium decreases. Therefore, most patients will require additional therapy. Children with milder forms of VDDR2a, such as those without alopecia, may have clinical and radiologic improvement with administration of high-dose vitamin D therapy that ranges from 5,000 to 40,000 IU per day of calciferol, 20–200 μg per day of calcifediol, or 17–20 mcg per day of calcitriol (38). Many patients will achieve a complete remission as long as they are receiving very high doses of calciferols (39). Among patients with alopecia, about half will be resistant to even the highest doses of calciferols; the other half have demonstrated satisfactory calcemic responses, but have required doses that are typically 10-times greater than in those with normal hair. Maintenance therapy is based upon four factors: (i) some patients will respond to calciferols [vitamin D_3_, vitamin D_2_, or 25(OH)D_3_] that are substrates for generation of 1,25(OH)_2_D; (ii) other patients may respond only to high doses of vitamin D analogs [1,25(OH)_2_D_3_ or 1α(OH)D_3_] that possess 1α-hydroxylation; (iii) a minority will not respond to calciferols at any dose.

Some patients will be unable to produce sufficient 1,25(OH)_2_D to overcome the VDR defect but nevertheless will respond to extraordinarily high doses of active forms of vitamin D [i.e., 1,25 (OH)_2_D_3_ or 1α(OH)D_3_]. These patients should receive consistent daily supplementation with calcium (1,000 mg per 24 h elemental calcium).

Those patients who fail to respond to maximal doses of calciferol will require intravenous infusions of calcium (1,000 mg elemental calcium per 24 h infused over 12 h), which can be used even by the youngest children with VDDR2a. Because normal calcium balance during childhood growth is about 300 mg/24 h, affected children often have large deficits prior to treatment. Hence, intravenous infusions of calcium must be administered over many months to achieve clinically meaningful results. Administration of high doses of oral calcium is usually unsuccessful because the upper limit of tolerance to oral calcium is about 6 g per day, and in the absence of vitamin D action the net calcium retention is only about 10%.

Remarkably, although fractional calcium absorption is low from early childhood through the end of puberty, during and after puberty subjects with VDDR2a develop an unsual adapation that is characterized by an increase in calcium absorption to levels that are even greater than those of normal subjects. Accordingly, many affected patients will be able to maintain normal plasma levels of calcium with more modest oral calcium supplementation or, at least in some cases, even without calcum supplements altogether. Many patients maintain near normal PTH concentrations and normal bone mineral density, although they continue to have elevated serum concentrations of 1,25(OH)_2_D_3_, suggesting persistent target organ resistance (40, 41).

## Vitamin D Dependent Rickets Type 2b (VDDR2b)

VDDR2b (OMIM 600785) resembles VDDR2a but is not caused by a defect in the *VDR* gene. Rather, the molecular defect appears to be overexpression of a nuclear protein that specifically interacts with a DNA response element that binds retinoid X receptor-VDR heterodimers. This dominant-negative protein appears to be a member of the family of heterogeneous nuclear ribonucleoproteins (hnRNPs), which attenuate gene transcription via their role as hormone response element-binding proteins. Chen et al. (42) have proposed that the vitamin D resistance present in VDDR2b is similar to that described in New World primates. These animals show abnormal expression of a hormone response element-binding protein that leads to target cell resistance to vitamin D as well as other steroid hormones. No gene has yet been identified as the cause of VDDR2b so it is impossible to state with precision the number of affected subjects. An early desription of VDDR2b identified a cluster of more than 200 affected children living in the Cauca Department in the southwest part of Colombia (43), but no additional information about these subjects is available. VDDR2b appears to be a very uncommon condition, if indeed it is a distinct disorder at all. Management of rickets is similar to that described above for VDDR2a.

## Vitamin D Dependency Rickets Type 3 (VDDR3)

VDDR3 is due to increased inactivation of vitamin D metabolites and is caused by a recurrent gain-of-function missense mutation (p.I301T) in the gene encoding CYP3A4 (44), the most abundant hepatic CYP450 enzyme. The clinical features appear similar to those of patients with VDDR1, but given the small number of reported cases of VDDR3 it may be premature to specify a definitive a description. The original report described two unrelated subjects (44). Proband 1 first presented before age 2 years with a medical history of genu varum and an unsteady gait. Biochemical assessment revealed reduced serum levels of calcium and phosphorus with increased serum levels of alkaline phosphatase and PTH. Proband 2 did not walk until 4.5 years of age and had been treated unsuccessfully for vitamin D deficiency using conventional dosages of vitamin D. Both children were short and demonstrated classical radiological features of rickets. Both affected individuals had detectable serum concentrations of vitamin D3 but low serum concentrations of 25(OH)D and 1,25(OH)_2_D that increased after administration of very large doses of vitamin D or calcitriol and which decreased rapidly thereafter. Both patients required high doses of calcitriol or vitamin D3 (50,000 IU daily) to maintain normal serum concentrations of vitamin D metabolites as well as normal serum levels of PTH, calcium, and phosphorus.

*In vitro* studies of the CYP3A4 p.I301T mutation shows that this enzyme rapidly and extensively inactivates vitamin D metabolites (45), which explains the poor response of affected children to normal doses of vitamin D or vitamin D analogs. These children do respond to parent vitamin D or vitamin D metabolites, but require far greater doses than are necessary in nutritional vitamin D deficiency. They require life long treatment with high dose vitamin D to maintain a biochemcial and clinical remission.

## Conclusions

The biochemical and genetic analyses of various forms of VDDR has yielded important insights into vitamin D homeostasis and 1,25(OH)2D action. Similarly, continued study of children and adults with VDDR has the potential to provide a more complete understanding of the biological role of vitamin D not only in bone and mineral metabolism but also in other, non-traditional vitamin D actions. The management of VDDR remains a challenge, particularly for treatment of patients with VDDR2, who are often completely resistant to vitamin D and its metabolites. Ongoing studies will be essential to promote the well-being of the families with VDDR and in improving the diagnostic and clinical management of these uncommon genetic disorders.

## Author Contributions

ML conceived the topic and wrote the content.

## Conflict of Interest

The author declares that the research was conducted in the absence of any commercial or financial relationships that could be construed as a potential conflict of interest.
